# PrP^C^ Promotes Endometriosis Progression by Reprogramming Cholesterol Metabolism and Estrogen Biosynthesis of Endometrial Stromal Cells through PPARα Pathway

**DOI:** 10.7150/ijbs.68015

**Published:** 2022-02-07

**Authors:** Hai-Yan Peng, Sha-Ting Lei, Shu-Hui Hou, Li-Chun Weng, Qing Yuan, Ming-Qing Li, Dong Zhao

**Affiliations:** 1 Shanghai Key Laboratory of Maternal Fetal Medicine, Clinical and Translational Research Center, Shanghai First Maternity and Infant Hospital, School of Medicine, Tongji University, Shanghai 200092, China; 2 Department of Obstetrics and Gynecology, Shanghai Ninth People' s Hospital, School of Medicine, Shanghai Jiao Tong University, Shanghai 200011, China; 3 Department of Gynecology, Shanghai First Maternity and Infant Hospital, School of Medicine, Tongji University, Shanghai 200092, China; 4 Laboratory for Reproductive Immunology, Shanghai Key Laboratory of Female Reproductive Endocrine Related Diseases, Obstetrics and Gynecology Hospital of Fudan University, Shanghai 200011, China; 5 Key Laboratory of Reproduction Regulation of NPFPC, SIPPR, IRD, Fudan University, Shanghai 200032, China

**Keywords:** endometriosis (EMs), prion (PrP^C^), cholesterol metabolsim, estrogen, PPARα

## Abstract

Endometriosis (EMs) is characterized as an estrogen-dependent disease. Whereas, the underlying mechanism for activated estrogen biosynthesis in EMs lesions is largely unknown. We analyzed cholesterol metabolism and estrogen biosynthesis condition of EMs lesions by biological information analysis of GEO datasets, and further verified both *in vitro* and* in vivo* by constructing EMs models with uterus fragments from donors of PRNP knockout mouse (*Prnp*^-/-^, KO119), Octapeptide repeat region of PRNP knockout mouse (KO120) and PRNP transgenic mouse (Tg20). We found that transcriptome of cholesterol metabolism and estrogen-converting enzymes were disturbed in EMs patients, and cellular cholesterol concentration and local estradiol level were substantially increased in EMs lesions, as well as the high level of prion (PrP^C^, encoded by PRNP). Notably, 17-β estradiol stimulation significantly up-regulated PrP^C^ expression in endometrial stromal cells (ESC) and PrP^C^ promoted the proliferative, migratory and invasive abilities of ESC, and was further verified to accelerate EMs progression in mouse models. More importantly, PrP^C^ promoted cholesterol accumulation and activated estrogen biosynthesis of ESC in a PPARα pathway-dependent manner. Taken together, this study suggests that PrP^C^-cholesterol metabolism/estrogen biosynthesis contributes to the progression of EMs by negatively regulating PPARα pathway, and could be potential therapeutic targets for EMs intervention.

## Introduction

Endometriosis (EMs) is a kind of benign gynecological disease, conceptualized as viable endometrial tissues survived outside the uterus cavity and prevalent in about 10% women of reproductive age 1, resulting in dysmenorrhea, chronic pelvic inflammation and even infertility, which serves as a cruel health killer to women worldwide 2-4. Studies have focused on uncovering EMs pathogenesis and multiple factors, especially augmented local estrogen biosynthesis, are found out to contribute to its progression 5-7. As we known, estrogen fundamentally impacts on fate of EMs loci, and endometriotic estradiol concentration is predominantly determined by local biosynhesis but not circulating levels, reported by Huhtinen K 8, implying that local estrogen biosynthesis is really important to EMs development. Increasing number of researches have concerned this issues in the past few years, it is reported that estrogen biosynthesis and production indeed take place in EMs lesions, for they possessing of integrated estrogen-converting enzyme system, and estrogen level localized in EMs loci definitely elevates and promotes lesion survival 9-12, with underlying mechanism remaining to be elucidated. Additionally, strategies targeted on local estrogen-converting enzymes and biosynthesis processes may facilitate developing new therapeutic approaches for EMs intervention 13-14. Cholesterol is a critical material for estrogen biosynthesis. In human body, cholesterol is mostly synthesized in liver and it is the main origin of cholesterol, other tissues have a weaker biosynthesis ability and contribute to local cellular cholesterol, which exerts effects in many biological processes. There exist 4 main factors that synthetically determine cellular cholesterol concentration, that are predominately rate-limiting enzyme HMG-CoA reductase (HMGCR)-mediated biosynthesis, ATP-binding cassette transporter type A1 (ABCA1)-regulated efflux, transport and acetyl-CoA acetyltransferase 1 (ACAT1)-mediated esterification processes 15. Generally, cellular cholesterol level is in a state of dynamic equilibrium and it could be disturbed in some pathological states. In recent years, cholesterol has emerged as a novel and essential mediator in regulating lipid metabolism, and its role in cancers and some other diseases has also been largely revealed 16-17. However, less research has paid attention to cholesterol metabolism and estrogen biosynthesis in EMs.

Prion (PrP^C^) is a kind of membrane glycosylphosphatidylinositol (GPI)-anchored proteins, widely expressed within the nervous system and well known to researchers by prion disease and bovine spongiform encephalopathy resulting from a pathological misfolded isoform of prion protein (PrP^SC^) 18-19. Cellular PrP^C^ regulates subtype of mesenchymal-like molecular, predicts colorectal cancer outcome 20, prevents several types of cancer cells from apoptosis induced by endoplasmic reticulum stress 21, and motivates gastric cancer cells invasion and metastasis 22. In reproductive system, the role of PrP^C^ is largely undefined, one research reports that PrP^C^ expression can be promoted by estrogen and hindered by progesterone in mouse uterus 23. Additionally, several studies discover a reciprocal connection between PrP^C^ and cholesterol metabolism in numerous pathophysiological processes, it is reported that cholesterol transporter ABCA1 affects PrP^C^ concentration by post-translational means in neuroblastoma cells 24, and conversion of PrP^C^ to PrP^SC^ affects cholesterol metabolism of neuronal cells 25, thus PrP^C^ may have a tight interaction with cholesterol metabolism in reproductive system. To identify this conjecture, our study focused on clarifying the transcriptome of cholesterol metabolism in normal and ectopic endometrial tissues by analyzing GEO datasets and disclosing the critical role of PrP^C^ in EMs development, hoping to provide more innovative therapeutic targets for EMs intervention.

## Materials and Methods

### Ethics statement

This study has been approved by the ethics committee of Shanghai First Maternity and Infant Hospital and all in vivo experiments have been approved by the Animal Care Committee of Tongji University.

### Cells culture

The normal immortalized human endometrial stromal cell line (HESC) was purchased from Cell Bank of Chinese Academy of Science (Shanghai, China) and authenticated by Genetic Testing Biotechnology Corporation (Suzhou, China) with STR profiling method. HESC and primary endometrial stromal cells (ESC) isolated from fresh specimens were all cultured by DME/F12 medium (Hyclone, USA), supplemented with 10% fetal bovine serum (FBS) (Sciencell, USA) and 1% antibiotics (NCE, China). The culture medium was exchanged every other day and cells were digested by 0.25% EDTA trypsin (NCE, China) when they grew up to 80%~90% confluence, then the digested cells were resuspended, planted in two sterile flask (Corning, USA) and cultured in incubator of 37℃, 5% CO_2_ atmosphere.

### Clinical specimens collection and primary endometrial stromal cells (ESC) isolation

This study enrolled 20 cases of tuber infertility (used as control patients) and 29 cases of ovarian EMs patients, all pariticipants with informed consent have not used hormonal drugs in the past 6 months, and EMs stage of patients were classified in accordance with revised American Society of Reproductive Medicine (ASRM) classification. Menstrual cycle phase of participants were all verified by histopathologists and the clinical characteristics of patients were presented in **Table 1** and **Table S1**. Normal endometrium and ovarian endometrial cystic wall were collected in sterile tubes during surgery procedure and immediately transferred to laboratory on ice, then samples were divided into three parts, one for isolating primary ESC, one was fixed by 4% formalin for immunohistochemistry assay, and another one was stored in -80℃ refrigerator for extracting RNA and protein later. For isolating ESC, endometrial specimens were dissected into tiny pieces with scissors and digested by type IV collagenase (Sigma, USA) for 30 minutes (min) at 37℃, then they were filtered through 70 μm filter (FALCON, USA) and centrifuged at 1000 rpm for 10 min, and the cells sediment were suspended by medium and cultured in 37℃, 5% CO_2_ atmosphere. Isolated ESC were verified beforehand by our research group through vimentin and cytokeratin 7 detection.

### Biological information analysis

To uncover the underlying transcriptome distinction between normal and ectopic endometrial tissues, we took advantage of biological information analysis and downloaded 9 datasets of different types of EMs (GEO accession: GSE23339, GSE7305, GSE25628, GSE12768, GSE78851, GSE58178, GSE87809, GSE47360 and GSE120103) from the NCBI Gene Expression Omnibus database (GEO), which were uploaded by researchers of relevant field worldwide and were almost RNA-sequencing data and covered huge amount of undiscovered biological information. We downloaded original data of all these 9 GEO datasets, and compared transcriptional expression levels of cholesterol biosynthesis, cholesterol efflux and transport related molecules, and estrogen-converting enzyme according to the platform annotation. All original data was statistically analyzed by GraphPad Prism version 6.0 software and the transcriptome profile of EMs were graphed.

### RNA extracting and quantitative real‑time polymerase chain reaction (qRT-PCR) assay

Groups of cells and dissected tissue samples were lysised by trizol (TAKARA, Japan) for 10 min on ice, and RNA was extracted by traditional methods with using of chloroform, isopropanol and ethanol, then the RNA was measured and tested for the quality, then qualified RNA was reversely transcripted to cDNA according to the manufacturer's instructions, which procedure cost about 15 min. The products could be reserved in -20℃ refrigerator temporarily or immediately being used in PCR process (Applied Biosystems), the primers constructed by Sangon Biotech Co., Ltd (**Table S2**), cDNA and other reagents (TAKARA, Japan) (**Table S3**) were mixed and reacted as instructed, the whole process last for about 1 hour (h), and the expression level of gene was determined by the comparative ΔΔCT method and *GAPDH* or *β-ACTIN* was used as internal control.

### Western blotting assay

We used pre-cooling RIPA (Beyotime, Shanghai) to treat the ESC and endometrial tissues for about 30 min, the lysis was collected in EP tube and centrifuged at 4℃, 12000 rpm for 20 min, then we abandoned the deposits and reserved the protein supernatants. Protein concentration of samples was quantified by BCA method (Beyotime), and then protein samples were diluted with loading buffer (Beyotime) and heated at 95℃ for 10 min. Upon the SDS-PAGE gel was prepared, 25 μg protein samples were added into the gel and underwent electrophoresis process, and molecules of different weights were separated in gel and further transferred to the PVDF membrane (Millipore, USA). We prepared 5% blocking reagent by resolving non-fat milk powder in TBST buffer (Biotech Well, Shanghai), and the PVDF membrane was blocked for 1.5 h at room temperature. After blocking process, the PVDF membranes were incubated in the diluted primary rabbit antibody at 4℃ overnight and washed with TBST for 3 times, then they were incubated in diluted HRP conjugated goat-anti-rabbit antibody for 1 h at room temperature. Lastly, the PVDF membranes were exposed with immobilon western chemilum HRP substrate (Millipore) and expression level of protein was analyzed according to relative density of bands.

### Flow cytometry assay (FCM)

Membrane and cellular PrP^C^ (CD230) in HESC and primary ESC was detected by flow cytometry assay, cells were digested by non-EDTA trypsin (NCM, China), washed by pre-cooling PBS (Hyclone) and centrifuged at 1000 rpm for 10 min, then cells were fixed and permed by buffer (DAKEWE, China) and cellular PrP^C^ was detected. Cells were incubated with PE-anti-CD230 antibody (DAKEWE) for 30 min at room temperature protected from light. After incubation process, the cells were washed by PBS and 400 μl of cell suspension samples were added into the flow cytometry for detection, and the intensity of fluorescence was used to determine PrP^C^ expression level.

### Immunohistochemistry (IHC)

The fresh normal endometrium and ectopic endometriotic lesions were fixed by 4% formalin overnight and underwent embedding and slicing process. Then the specimens were roasted in oven for 2 h at 65℃ and immersed in density gradient methylbenzene and ethyl alcohol for dewaxing and dehydration purposes. We further treated the specimens with antigen retrieval reagent, and incubated the specimens in diluted primary rabbit-anti-Prion antibody (Abcam, USA) at 4℃ overnight. The next day, the antibody was recycled and specimens were washed by PBS for 3 times and incubated in diluted HRP-conjugated goat-anti-rabbit antibody for 30 min at room temperature, then the specimens were reacted with DAB horseradish peroxidase color development kit (Biotech Well, Shanghai) for about 30 min and then dyed by hematoxylin. At last, the slides were blocked by neutral resins and we observed and photographed them under microscope. The score of slides were determined according to the positively stained area: no positively stained cell scored 0; positively stained area less than 10% scored 1; positively stained area of 11%~50% socred 2 and scored 3 when positively stained area reached 51% or more.

### Enzyme-linked immunosorbent assay (ELISA)

The supernatants and lysis of ESC were collected after cells being cultured for 24 h or 48 h, and protein which extracted from cells and tissues, were preserved after being determined of concentration by BCA method (Beyotime). 17 beta-estradiol ELISA kit (IBL, Germany) was taken out of refrigerator and warmed to room temperature, then standard samples, supernatants and cells lysis were added into the 96-well detection plate, and antigens could react with coated antibody for hours according to the manufacturer's instructions. After undergoing a series of procedures, the detection plate was measured by microplate reader at optical density of 420 nm, and the concentration of 17 beta-estradiol of all samples were determined by optical values referred to standard curve.

### Quantification of cellular cholesterol level

The cellular cholesterol level in our study was measured by two methods, one was detected by cholesterol assay kit (cell-based) (Abcam) to inspect the intensity of fluorescence under the ultraviolet light, the other was determined by Red Cholesterol Assay Kit (Invitrogen, USA). Simply, the former methods could be described as: cells being fixed by fixation buffer for 10 min and washed for 3 times by wash buffer, then cells were stained for Filipin III and inspected under fluorescence microscope. In the latter method, cells were lysised by RIPA and measured by BCA method (Beyotime), then samples were mixed and reacted with reagent of Red Cholesterol Assay Kit for 30 min in black 96-well plate, and lastly the plate was detected by microplate reader using excitation at 540 nm and emission detection at 590 nm, and the concentration of cellular cholesterol was determined according to standard curve and standardized by protein concentration.

### Lentivirus construction and infection

Lentivirus of negative control (CON077) and LV-PRNP-RNAi (hU6-MCS-Ubiquitin-EGFP-IRES-puromycin), negative control (CON335) and LV-PRNP (Ubi-MCS-3FLAG-CBh-gcGFP-IRES-puromycin) were constructed by GENECHEM Biotech Co., Ltd. The HESC were digested and uniformly plant in 6-well plate, when the cells were cultured for 24 h, attached to the bottom of flask and grew to confluence of 50%, lentivirus and infection enhance reagent were added into the culture medium, and we exchanged the medium after 12 h of infection. 72 h later, the cells were inspected under the fluorescence microscope and analyzed by qRT-PCR or western blotting assay for evaluating the infection efficiency, then the infected cells were treated by puromycin for 7 days to select out the successfully infected cells.

### CCK8 cell viability, cell migration and invasion assay

For cell viability detection, the cells were digested and planted in 96-well plate, treated and cultured for 24 h or 48 h, and then the Cell Counting Kit-8 (CCK8) (DOJINDO, Japan) reagent was diluted by medium, with the ratio of 1 part of CCK8 diluting with 9 parts of medium. The diluted CCK8 reagent was added into the 96-well plate and incubated at 37℃ for 30 min protected from light, then the flask was measured by microplate reader at optical density of 450 nm. For measuring cell migration and invasion ability, medium supplemented with 20% FBS was added into the bottom of 24-well plate and each 10^5 cells suspended in 100 μl medium including 2% FBS were placed into the transwell (Corning, USA), and transwell for cell invasion assay was covered with diluted matrigel (BD, USA), then the plate was incubated in 37℃, 5% CO_2_ incubator. 24 h or 48 h later, we took out the transwell and wiped out the cell suspension inside, used 4% paraformaldehyde to treat cells for 10 min, and then dyed the outside bottom of transwell with crystal violet reagent (Biotech Well) for 30 min. Lastly, the transwell was washed by PBS and inspected under the microscope and randomly taken photos of 4 sights.

### The next RNA-sequencing

To disclose the transcriptome of PrP^C^ overexpressed ESC, the CON335 and LV-PRNP stable infected HESC were cultured in flask, when they grew up to 90% confluence, medium was wiped out and trizol (TAKARA) was added into the flask to lysis the cells, and RNA of two groups of cells was extracted by traditional extraction method, with triplicate samples for each group. We determined the concentration and quality of RNA, and equivalent qualified RNA samples were added into well and sequenced. When the sequence procedure finished, we downloaded the original data and verified whether the detection was valid, then biological information analysis such as KEGG pathway and GO enrichment was performed.

### Mass spectrometry analysis

The stable lentivirus infected cell lines CON335 and LV-PRNP HESC with transfection efficiency verified were cultured, with each group of cells having 6 repeated samples. When each sample grew up to quantity of 10^7 cells, they were digested by 0.25% EDTA trypsin (NCM), centrifuged at 1000 rpm for 10 min, then we abandoned the supernatants and cell deposits were treated by liquid nitrogen for 30 seconds (sec) and immediately transferred to BIOTREE Biological Technology Company (Shanghai) on dry ice. The samples were used for UHPLC-QTOF-MS detection after being qualified and expression levels of metabolites were determined, then differently expressed metabolites were further analyzed by bioinformatics analysis.

### Animal experiments

PRNP knockout mouse (*Prnp*^-/-^, KO119) and Octapeptide repeat region of PRNP knockout mouse (KO120) of C57BL/6J (C57) background, PRNP transgenic mouse (Tg20) and wild type (WT) mouse of C57BL/S129 background were all constructed by BRL Medicine Company, fed and bred in SPF environment and generously donated by Professor Yu-Lan Zhao of East China Normal University. To construct syngeneic mouse EMs models, 6~8 week (w) donor and recipient female mice were received intramuscular injection of 3 μg/mouse 17-β estrogen (Sigma, USA) at day 1 and day 4, then the uterus of donor mice were dissected into small pieces and intraperitoneally injected into the recipient mice at day 7, then the uterus debris planted in periotneal cavity of recipient mice and EMs models were constructed. In the following days, the EMs models were fed normally or intraperitoneally injected with HMGCR inhibitor pravastatin (10 mg/kg/day) (MCE, China) or mixture liquid of PBS and DMSO as control for 10 days, then EMs models were sacrificed and the ectopic lesions in periotneal cavity were collected at day 21. For collecting retroorbital blood samples, mice were synthesized and we collected blood from the right retroorbital plexus, all samples were centrifuged at 3000 rpm for 20 min and the plasma was reserved.

### Statistical analysis

Research data in this manuscript were all exhibited as mean ± standard error of the mean (SEM). Student's t-test, one-way analysis of variance (ANOVA) and Mann-Whitney test were taken advantage of making statistical analysis by the GraphPad Prism version 6.0 software, and *P* value less than 0.05 was considered as significant difference.

## Results

### EMs lesions exhibit as state of cholesterol accumulation and activated local 17-β estradiol biosynthesis

**Figure 1:**
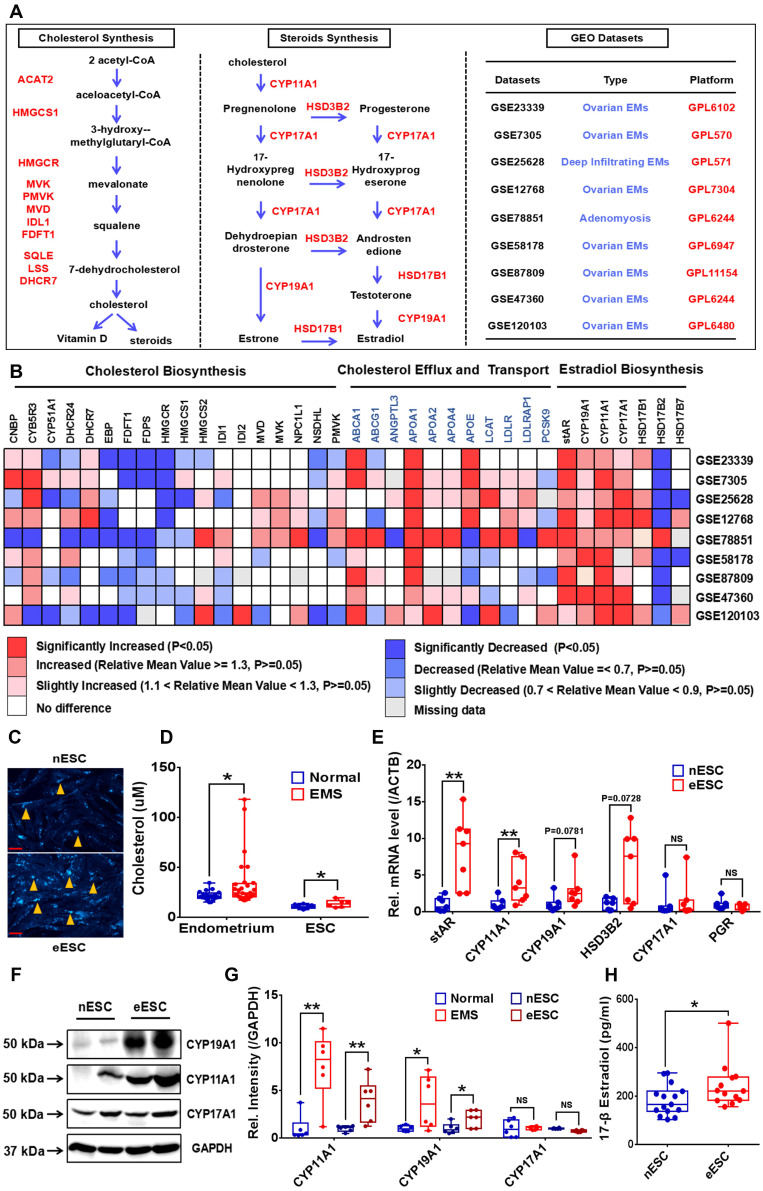
** EMs lesions exhibit as state of accumulating cholesterol and activated estradiol biosynthesis.** (A-B) Transcriptomes of cholesterol metabolism (cholesterol biosynthesis, transport and efflux) and estradiol biosynthesis related genes were analyzed in 9 EMs related GEO datasets (GSE23339, GSE7305, GSE25628, GSE12768, GSE78851, GSE58178, GSE87809, GSE47360 and GSE120103). (C) Determination of cellular cholesterol in primary ESC. (D) Cholesterol concentration increased in lysis of EMs lesions (n=29) and eESC (n=6), when compared to normal endometrial tissues (n=20) and nESC (n=10). (E) Expression level of *STAR*, *CYP11A1*, *CYP19A1* and *HSD3B2* elevated in eESC, and expression of *CYP17A1* and *PGR* had no significant difference between normal and ectopic ESC. (F) Protein level of CYP19A1, CYP11A1 and CYP17A1 were detected by western blotting assay in endometrium tissues and ESC. (G) Protein level of CYP19A1 and CYP11A1 increased in EMs lesions and eESC, with no significant difference in CYP17A1 level. (H) Concentration of 17-β estradiol augmented in supernatants of eESC (n=13), when compared to nESC (n=15). Data was showed as mean ± SEM, Student's t-test, one-way analysis of variance (ANOVA) and Mann-Whitney test were used to make statistical analysis. *P<0.05, **P<0.01, NS: no significant difference. EMs: endometriosis, GEO: Gene Expression Omnibus, ESC: endometrial stromal cells, nESC: normal endometrial stromal cells, eESC: ectopic endometrial stromal cells. Red scale bar: 30 μm. Orange arrow: indicating cellular cholesterol.

To uncover the cholesterol metabolism and local estrogen biosynthesis condition in EMs lesions, we profiled transcriptome of cholesterol metabolism and estrogen-converting enzymes in 9 GEO datasets of EMs (GEO accession: GSE23339, GSE7305, GSE25628, GSE12768, GSE78851, GSE58178, GSE87809, GSE47360 and GSE120103), results showed that transcription of cholesterol efflux (e.g, *ABCA1*), cholesterol transport (e.g, *APOA1*, *APOA2* and *APOA4)* and steroid biosynthesis related enzymes coding genes (e.g, *STAR*, *CYP11A1*, *CYP17A1* and *CYP19A1*) were activated in ectopic lesions, and expression level of cholesterol biosynthesis related genes (e.g, *HMGCR*, *EBP*, *FDFT1* and *FDPS*) were hindered, and *in vitro* experiments consistently showed that protein expression level of HMGCR in eESC decreased (**Figure 1A-1B, Figure S1A**), indicating that cholesterol metabolism and estrogen biosynthesis in EMs lesions were significantly disturbed. Further analysis showed that cellular cholesterol concentration substantially augmented in ectopic lesions and ESC (**Figure 1C-1D**). In addition, both gene and protein expression level of estrogen biosynthesis related enzymes (stAR, CYP11A1, CYP19A1 and HSD3B2) were significantly enhanced in ectopic lesions and ESC, which was consistent with results analyzed in GEO datasets (**Figure 1E-1G**). Furthermore, 17-β estradiol level in supernatants of ectopic ESC substantially elevated, when compared to normal ESC (**Figure 1H**), implying that local estrogen level augmented in EMs lesions.

### Estrogen-mediated PrP^C^ augmentation promotes ESC survival and accelerates EMs progression

**Figure 2:**
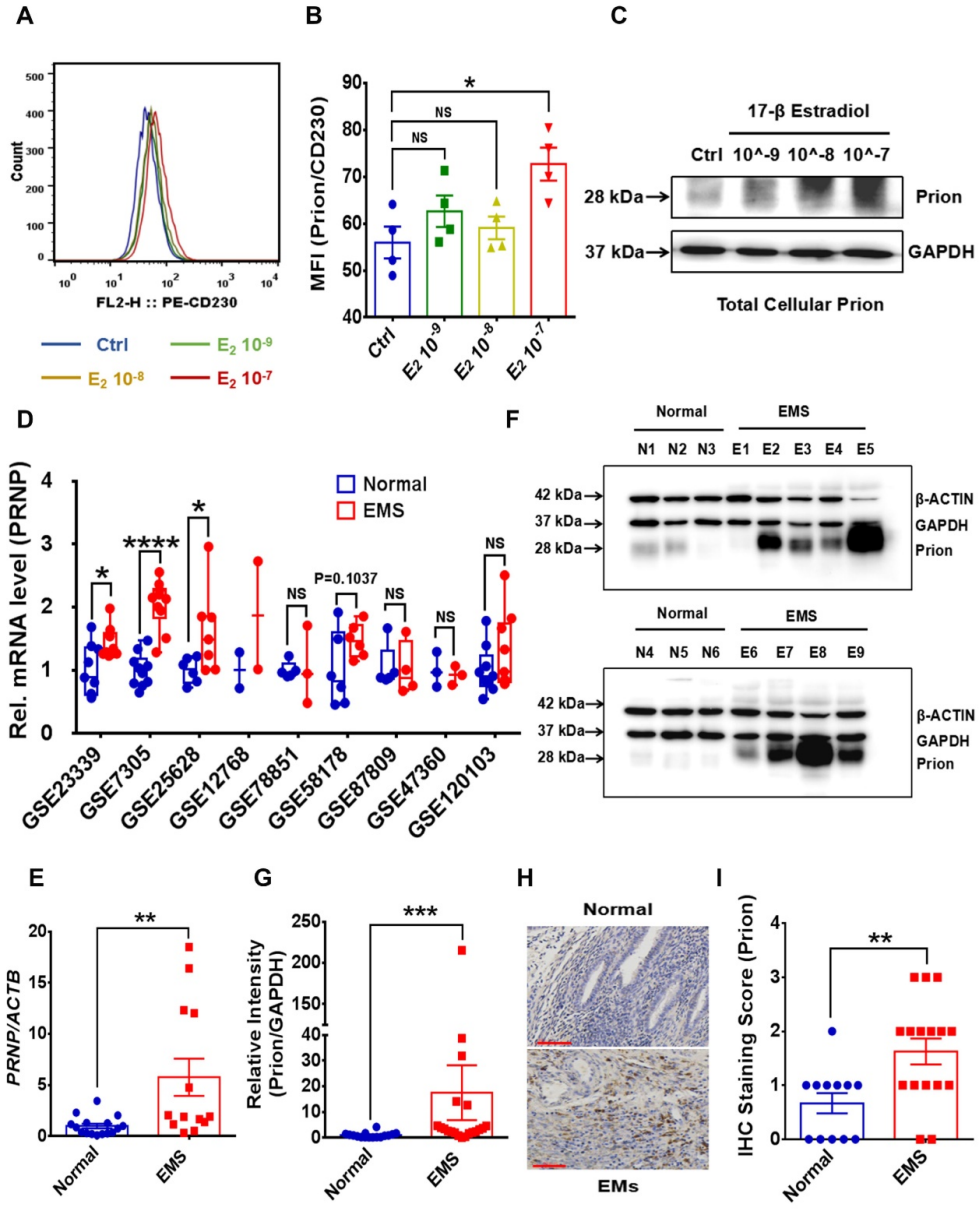
** Estrogen stimulation up-regulates PrPC expression and PrPC level is increased in EMs.** (A-C) Expression level of PrP^C^ in HESC was determined by FCM and western blotting assays, and high concentration of 17-β estradiol treatment promoted PrP^C^ expression in HESC. (D) Gene expression level of *PRNP* was analyzed in 9 EMs related GEO datasets and level of *PRNP* augmented in EMs lesions. (E) Gene expression level of *PRNP* was detected by qRT-PCR assay and increased in EMs endometrial tissues. (F) Protein level of PrP^C^ was detected in endometrial tissues by western blotting assay. (G) Relative protein level of PrP^C^ augmented in EMs lesions (n=20), when compared to normal endometrial tissues (n=16). (H-I) IHC assay revealed that PrPC level enhanced in EMs lesions (n=16), when compared to normal endometrial tissues (n=12), and mostly expressed in stromal tissues. Data was showed as mean ± SEM and Student's t-test, one-way analysis of variance (ANOVA) and Mann-Whitney test were used to make statistical analysis. *P<0.05, **P<0.01, ***P<0.001, ****P<0.0001, NS: no significant difference. E2: 17-β estradiol, 10^-9, 10^-8 and 10^-7 refer to 10^-9^ mol/L, 10^-8^ mol/L and 10^-7^ mol/L respectively. HESC: human endometrial stromal cells line, IHC: immunohistochemical staining, FCM: flow cytometry assay, qRT-PCR: quantitative real- time PCR assay. Red scale bar: 60 μm.

**Figure 3:**
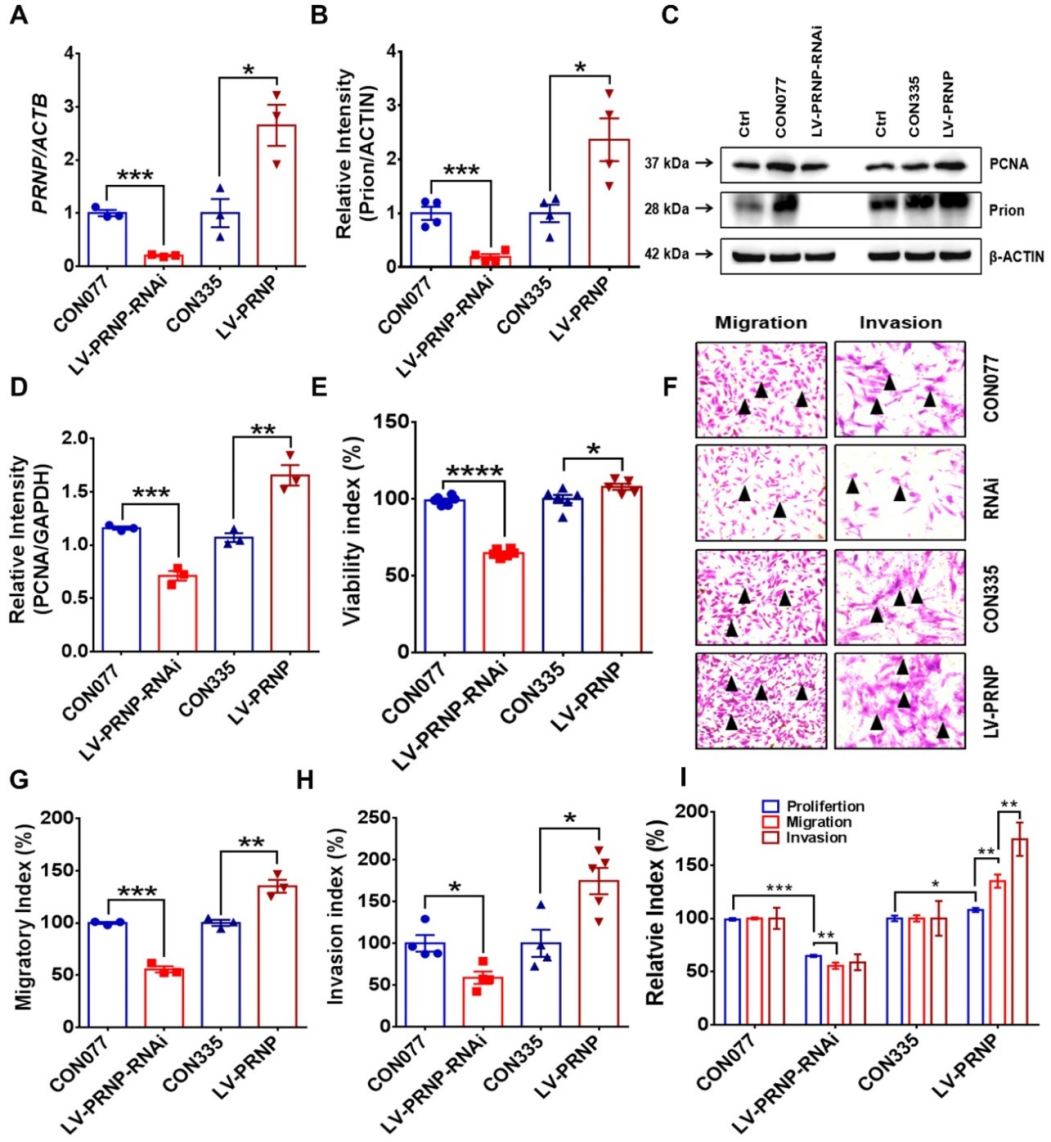
** Up-regulating expression of PrP^C^ promotes ESC proliferation, migration and invasion *in vitro*.** (A) Infection efficiency was determined by qRT-PCR assay. (B-C) Level of PrP^C^ was detected by western blotting assay and expression of PrP^C^ elevated in *PRNP* overexpressed ESC. (D) Up-regulating expression of PrP^C^ promoted PCNA expression. (E-H) Up-regulating expression of PrP^C^ enhanced proliferation, migration and invasion abilities of ESC. (I) Relative index of proliferation, migration and invasion of 4 groups of ESC and up-regulating expression of PrP^C^ strengthened migration and invasion ability of ESC more observably than promoting proliferation. Data was showed as mean ± SEM, Student's t-test, one-way analysis of variance (ANOVA) and Mann-Whitney test were used to make statistical analysis. *P<0.05, **P<0.01, ***P<0.001, ****P<0.0001. Black arrow: indicating ESC.

**Figure 4:**
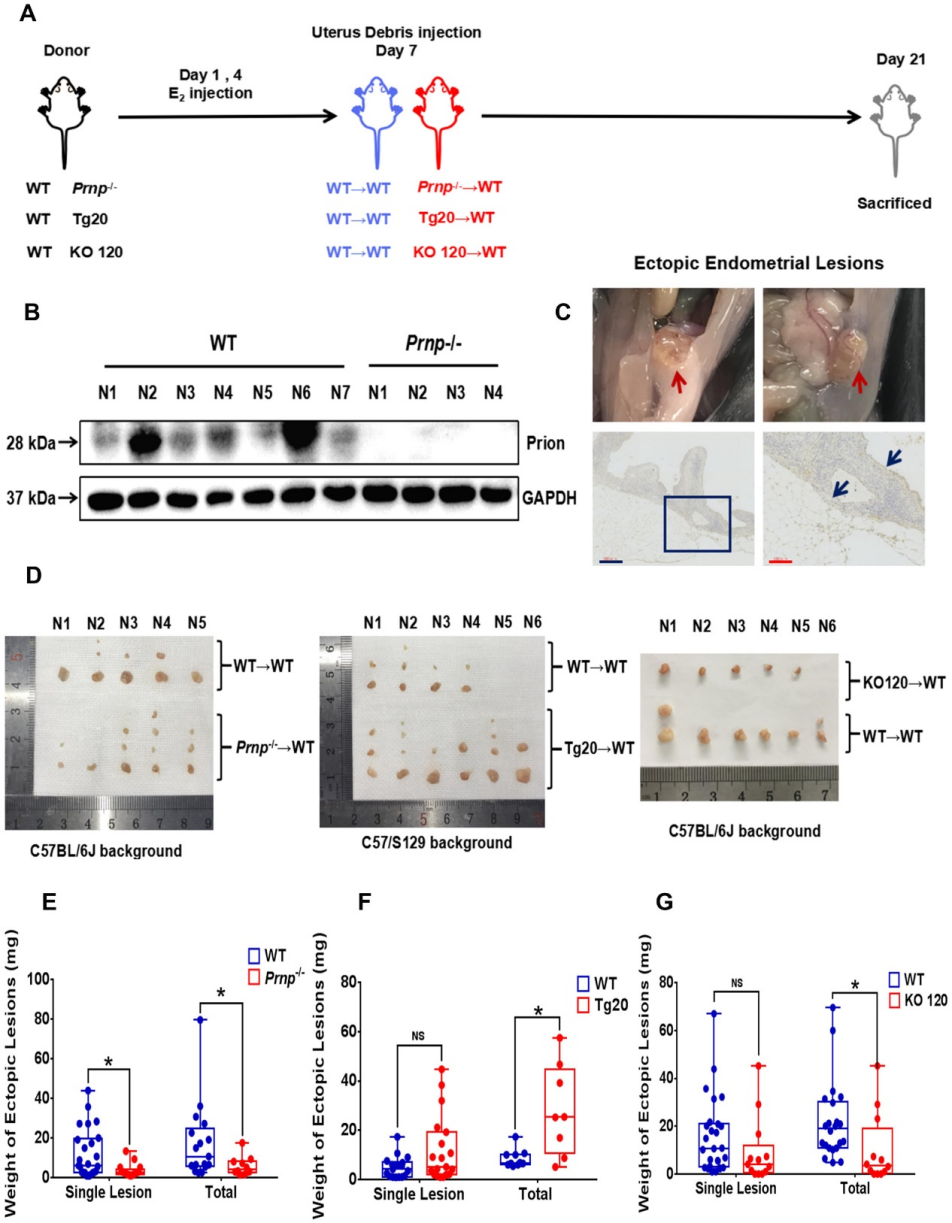
** PrP^C^ promotes EMs progression in mouse models.** (A) Ideograph for constructing EMs mouse models. (B) Levels of PrP^C^ were determined in mouse uterus specimens by western blotting assay. (C) Ideograph of ectopic lesions in mouse peritoneal cavity and histopathological examination results. (D) Collected ectopic lesions of EMs mouse models. (E) Weight of ectopic lesions decreased in *Prnp*-/- EMs mouse models (n=10), when compared to WT EMs models (n=17). (F) Weight of ectopic lesions augmented in Tg20 Ems mouse models (n=8), when compared to WT EMs models (n=10). (G) Weight of ectopic lesions diminished in KO120 EMs mouse models (n=12), when compared to WT EMs models (n=22). Data was showed as mean ± SEM, Student's t-test, one-way analysis of variance (ANOVA) and Mann-Whitney test were used to make statistical analysis. *P<0.05, NS: no significant difference. WT: wild type, *Prnp*-/-: PRNP knockout homozygote mouse (KO119), Tg20: PRNP overexpressed transgenic mouse, KO120: octopeptide repeat region of *PRNP* knockout mouse. Red arrow: ectopic lesions. Blue arrow: glandular epithelium of ectopic lesions in mouse models. Blue scale bar: 300 μm; Red scale bar: 100 μm.

To disclose potential relationship between PrP^C^ and activated local estrogen biosynthesis, estrogen stimulation was performed to ESC *in vitro*, and it displayed that estrogen treatment essentially up-regulated PrP^C^ expression in a concentration-dependent manner (**Figure 2A-2C**). In addition, biological information analysis by GEO datasets and clinical specimens collected from EMs and normal volunteers verified that PrP^C^ might be involved in the pathogenesis of EMs, for both gene and protein levels of PrP^C^ substantially elevated in ectopic lesions (**Figure 2D-2G**). Besides, IHC assay revealed that level of PrP^C^ in endometrial tissues was obviously uneven and preferentially expressed in stromal cells (**Figure 2H-2I**). To uncover the biological functions of PrP^C^ in EMs, HESC were infected with lentivrus and expression level of PrP^C^ stably changed (**Figure 3A-3C**), and PrP^C^ substantially promoted ESC survival by enhancing viability, migratory and invasive abilities of HESC, as well as up-regulating PCNA expression (**Figure 3D-3H**). Moreover, PrP^C^ had profound impacts on enhancing migration and invasion abilities of ESC independent of proliferation, for relative index of ESC migratory and invasive changed more observably than viability when expression of PrP^C^ was altered (**Figure 3I**). More importantly, *Prnp*^-/-^, KO120, Tg20 and WT mice were used as donors to construct EMs models, and ectopic lesions collected from these models displayed that *Prnp*^-/-^ group had attenuated and less weight of ectopic lesions, and disease severity aggravated in Tg20 group for they preserved more weight of ectopic lesions when compared to the WT group (**Figure 4A-4F**), meanwhile, weight of ectopic lesions in KO120 group of EMs models also increased (**Figure 4D, 4G**), implying that PrP^C^ accelerated EMs progression* in vivo*.

### PrP^C^ aggravates EMs severity by activating cholesterol accumulation and estrogen biosynthesis, and cholesterol lowering drug pravastatin attenuates EMs severity

**Figure 5:**
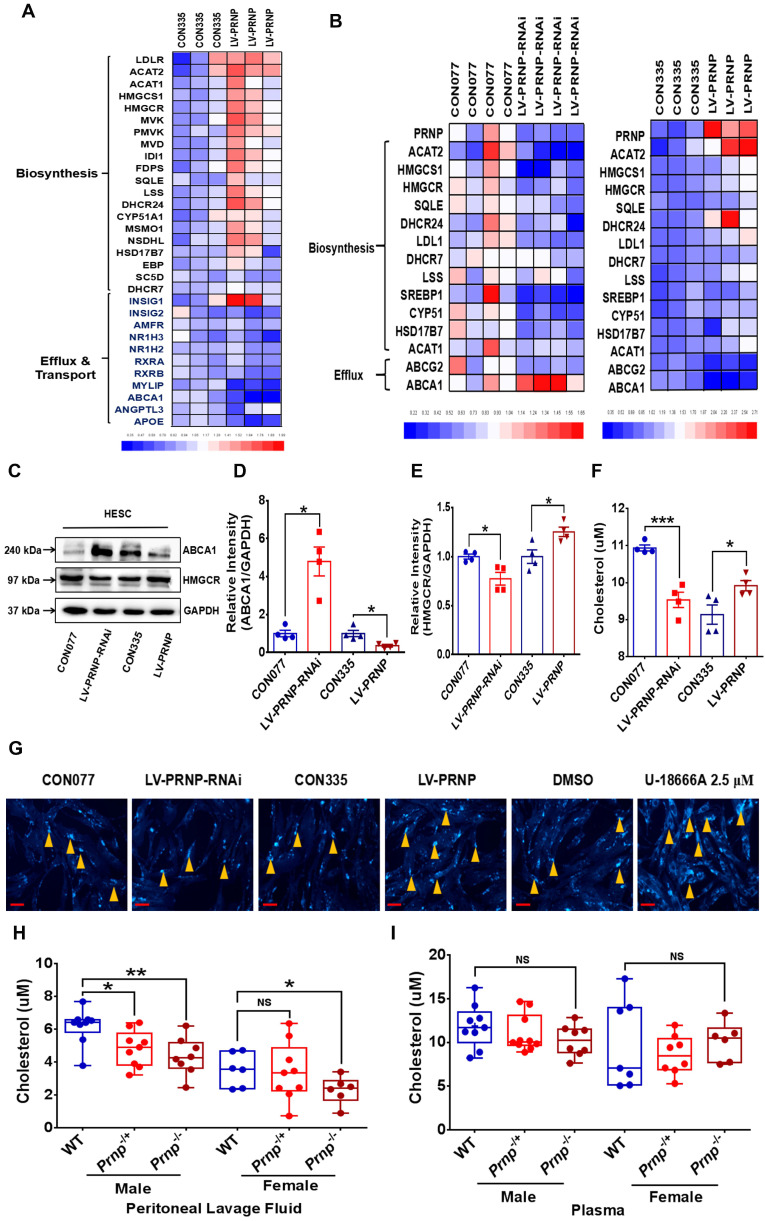
** PrP^C^ activates cellular cholesterol accumulation.** (A) The next RNA-Sequencing assay revealed increased cholesterol biosynthesis and inhibited cholesterol efflux and transport in PrP^C^ overexpressed ESC. (B) Gene expression level of cholesterol biosynthesis and efflux related genes were determined by qRT-PCR assay in CON077, LV-PRNP-RNAi, CON335 and LV-PRNP ESC. (C-E) Western blotting assay exhibited that PrP^C^ promoted HMGCR and hindered ABCA1 expression in ESC. (F) Cellular cholesterol concentration augmented in PrPC overexpressed ESC. (G) Cellular cholesterol in 6 groups of ESC and U18666A treatment group was used as a positive control. (H) Cholesterol concentration was hindered peritoneal lavage fluid of both female and male *Prnp*-/- mouse (male: WT n=9, *Prnp*-/+ n=9, *Prnp*-/- n=8; female: WT n=6, *Prnp*-/+ n=9, *Prnp*-/- n=6), when compared to WT mouse. (I) No significant difference of cholesterol concentration was detected in plasma of WT and *Prnp*-/- mouse (male: WT n=9, *Prnp*-/+ n=10, *Prnp*-/- n=8; female: WT n=7, *Prnp*-/+ n=8, *Prnp*-/- n=6). Data was showed as mean ± SEM, Student's t-test, one-way analysis of variance (ANOVA) and Mann-Whitney test were used to make statistical analysis. *P<0.05, **P<0.01, NS: no significant difference. U18666A: cholesterol transport inhibitor. Red scale bar: 30 μm. Orange arrow: indicating cellular cholesterol.

In order to figure out the underlying mechanism behind PrP^C^ aggravating EMs severity, the next RNA-Sequencing assay was performed between control (CON335) and PRNP overexpressed (LV-PRNP) HESC, and we found out that transcription of cholesterol metabolism related genes significantly changed, with expression of cholesterol biosynthesis related genes (e.g. *HMGCR*, *HMGCS1*, *MVK* and *FDPS*) being activated and level of cholesterol efflux (e.g. *ABCA1*) and transport (e.g. *RXRA* and *APOE*) related genes being inhibited (**Figure 5A**). We further verified the results of RNA-Sequencing and role of PrP^C^ in reprogramming cellular cholesterol metabolism *in vitro*, and it displayed that up-regulating PrP^C^ expression elevated HMGCR level and hindered cholesterol efflux through depressing ABCA1 expression, further promoted cholesterol biosynthesis, and down-regulating PrP^C^ had an adverse effect, all had no influence on cholesterol esterification, for level of *ACAT1* and *ACAT2* having no significant difference (**Figure 5B-5E**). Being in consistent with the expression level of genes relating with cholesterol metabolism, cellular cholesterol concentration substantially increased in PRNP overexpressed HESC, with lessened cholesterol in PRNP down-regulated HESC (**Figure 5F-5G**). Moreover, comparing to WT mice, cholesterol concentration in peritoneal lavage fluid decreased in both male and female *Prnp*^-/-^ mice, with no significant difference in plasma (**Figure 5H-5I**).

**Figure 6:**
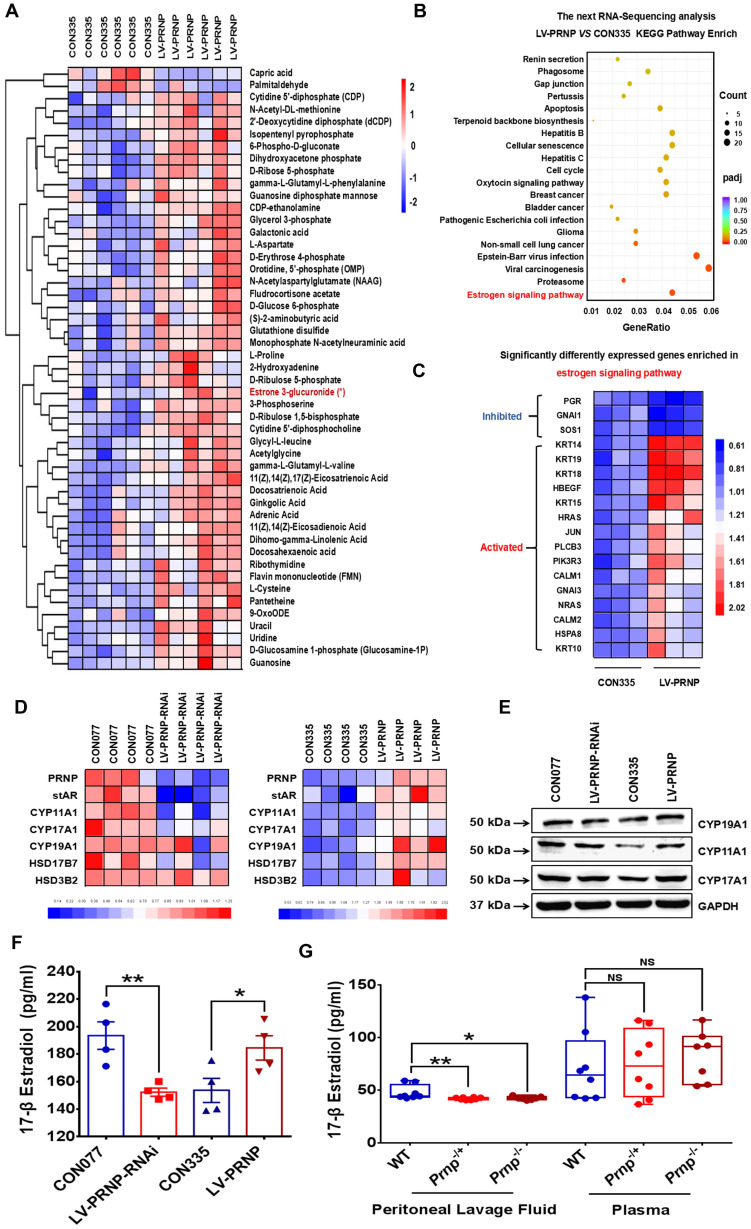
** PrP^C^ accelerates estradiol biosynthesis both *in vitro* and *in vivo*.** (A) Metabonomics detection displayed increased level of estrone in *PRNP* overexpressed ESC. (B-C) KEGG pathway analysis of the RNA-Sequencing results showed that estrogen signaling pathway was activated in *PRNP* overexpressed ESC. (D) Up-regulating expression level of *PRNP* promoted gene expression level of estradiol biosynthesis related enzymes (*stAR*, *CYP19A1*, *CYP11A1*, *CYP17A1*, *HSD17B7* and *HSD3B2*). (E) Protein levels of CYP19A1, CYP11A1 and CYP17A1 elevated in *PRNP* overexressed ESC. (F) PrP^C^ promoted 17-β estradiol biosynthesis in ESC. (G) Concentration of 17-β estradiol diminished in peritoneal lavage fluid of *Prnp*-/+ and *Prnp*-/- mouse, when compared to WT mouse and 17-β estradiol concentration had no significant difference in plasma of 3 groups of mouse. Data was showed as mean ± SEM, Student's t-test, one-way analysis of variance (ANOVA) and Mann-Whitney test were used to make statistical analysis. *P<0.05, **P<0.01, NS: no significant difference.

**Figure 7:**
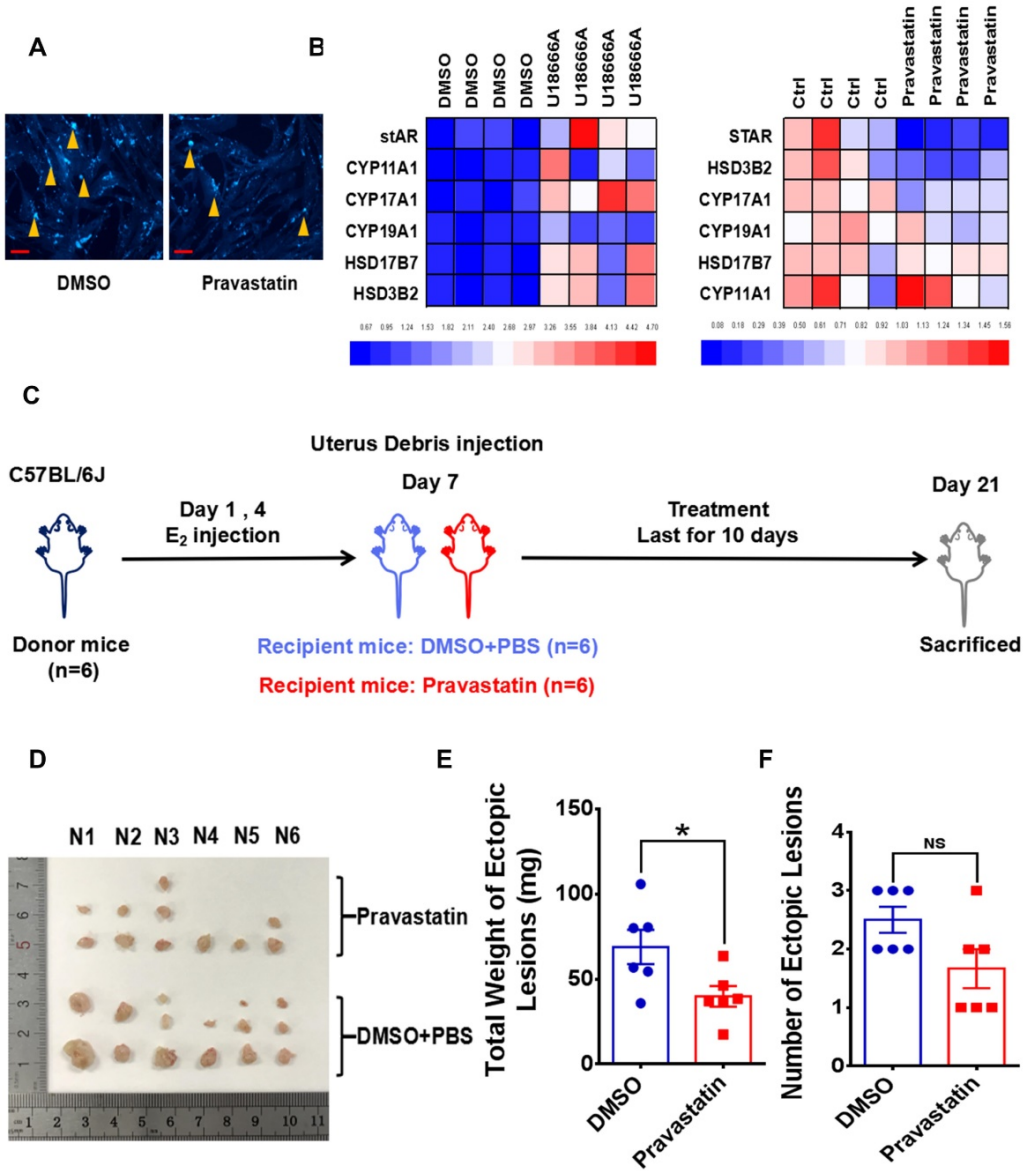
** Cholesterol lowering drugs pravastatin attenuates EMs severity in mouse models.** (A) Pravastatin treatment (1.0 μM) diminished cellular cholesterol level of ESC. (B) Cholesterol transport inhibitor U18666A (2.5 μM) stimulation promoted transcription of estradiol biosynthesis related genes (*stAR*, *CYP19A1*, *YP11A1*, *CYP17A1*, *HSD17B7* and *HSD3B2*) and pravastatin treatment (1.0 μM) showed adverse effects. (C) Ideograph for constructing EMs models and treatment strategies. (D) Ectopic lesions collected from DMSO and pravastatin (10 mg/kg/day) treatment EMs models. (E-F) Weight of ectopic lesions decreased in pravastatin treatment mouse models, and number of ectopic lesions had no significant difference between DMSO (n=6) and pravastatin treatment group (n=6). Data was showed as mean ± SEM, Student's t-test, one-way analysis of variance (ANOVA) and Mann-Whitney test were used to make statistical analysis. *P<0.05, NS: no significant difference. Red scale bar: 30 μm. Orange arrow: indicating cellular cholesterol.

Since cellular cholesterol is essential material for estrogen biosynthesis, we paid attention to the potential role of PrP^C^ in regulating local estrogen biosynthesis. Mass spectrometry analysis showed that cellular estrone-3 level increased in PRNP overexpressed HESC (**Figure 6A**), and KEGG pathway enrich analysis based on RNA-Sequencing results which was verified *in vitro* displayed that estrogen signaling pathway was activated (**Figure 6B-6C, Figure S1B**), implying that estrogen biosynthesis was active and vibrant in PrP^C^ up-regulated ESC. We determined both gene and protein expression levels of estrogen biosynthesis related enzymes, and it turned out that their levels substantially elevated in PRNP overexpressed ESC, with PRNP down-regulated ESC showing adverse effects (**Figure 6D-6E**). Furthermore, cellular concentration of 17-β estradiol increased in PRNP up-regulated ESC, and its level obviously decreased in PRNP down-regulated ESC and peritoneal lavage fluid of *Prnp*^-/+^ and *Prnp*^-/-^ mice, with no significant difference in plasma (**Figure 6F-6G**), indicating that PrP^C^ is important to reprogramme estrogen biosynthesis. In addition, cholesterol transport inhibitor U18666A and HMG-CoA reductase inhibitor, cholesterol lowering drug pravastatin were used to treat ESC and expression of estrogen biosynthesis related enzymes were determined, and it showed that U18666A-mediated accumulating cellular cholesterol in ESC promoted estrogen biosynthesis, and pravastatin had an adverse effect (**Figure 7A-7B**), indicating that cellular cholesterol level directly impacts on estrogen biosynthesis process. Based on these facts, we wondered whether pravastatin could be used as an approach to combat EMs, and it showed that intraperitoneal intervention of pravastatin significantly attenuated EMs severity (**Figure 7C-7F**).

### PrP^C^ reprograms cholesterol metabolism and estrogen biosynthesis through negatively regulating PPARα pathway

**Figure 8:**
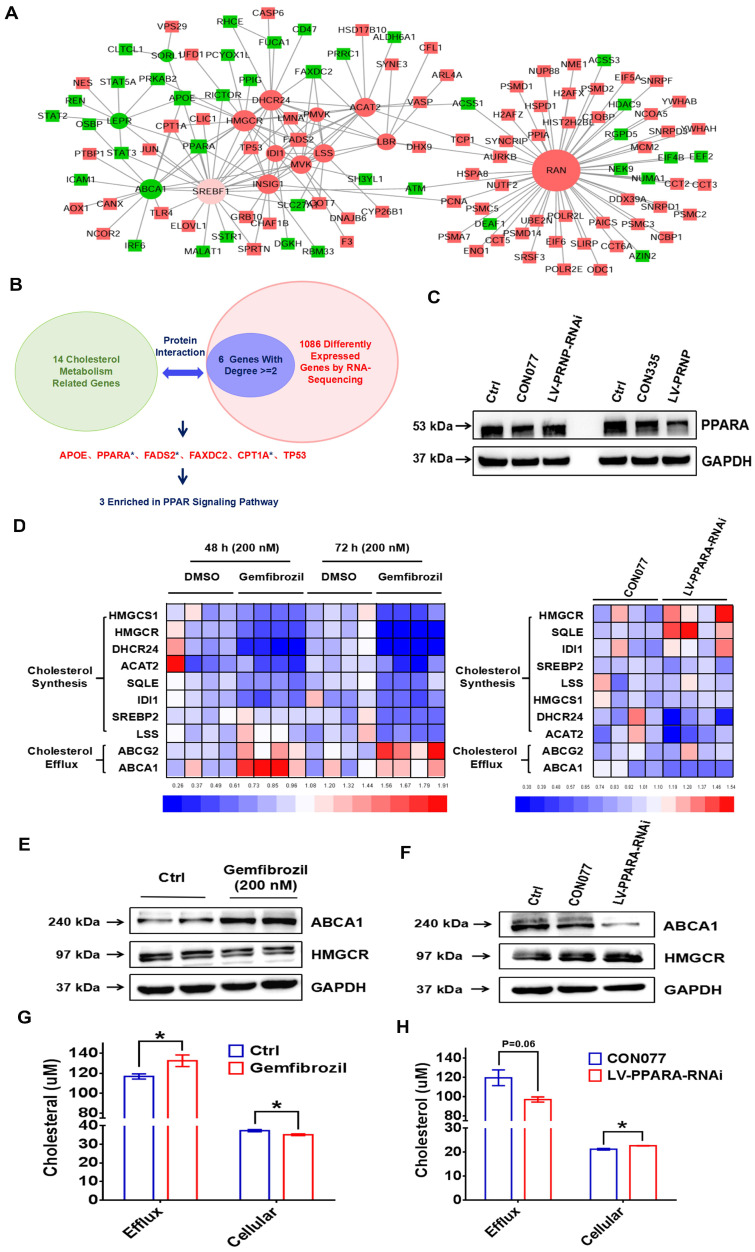
** PrP^C^ promotes EMs progression by reprogramming cholesterol metabolism and estrogen biosynthesis through PPARα pathway.** (A-B) Protein interaction analysis was performed between 14 cholesterol metabolism related genes (*ABCA1, SORL1, LEPR, DHCR24, ACAT2, HMGCR, IDI1, PMVK, LBR, RAN, MVK, LSS, INSIG1* and *SREBF1*) and 1086 different expressed genes of the next RNA-Sequencing, 6 genes (*APOE*, *PPARA*, *FADS2*, *FAXDC2*, *CPT1A* and *TP53*) with related degree being equal to or greater than 2 were selected out and 3 of them (*PPARA*, *FADS2* and *CPT1A*) were enriched in PPAR signalling pathway. (C) PrPC hindered expression level of PPARα. (D) Agonist of PPARα gemfibrozil (200 nM) treatment down-regulated cholesterol biosynthesis related genes level (*HMGCR*, *HMGCS1*, *DHCR24*, *ACAT2*, *SQLE*, *IDI1*, *SREBP2* and *LSS*) and promoted cholesterol efflux related genes expression (*ABCG2* and *ABCA1*). (E) Gemfibrozil treatment (200 nM) promoted ABCA1 and hindered HMGCR expression. (F) Down-regulating expression of PPARα hindered ABCA1 and promoted HMGCR expression in ESC. (G) Gemfibrozil (200 nM) treatment promoted cholesterol efflux and decreased cellular cholesterol level in ESC. (H) Down-regulating expression of PPARα decreased cholesterol efflux and enhanced cellular cholesterol level in ESC. Data was showed as mean ± SEM, Student's t-test, one-way analysis of variance (ANOVA) and Mann-Whitney test were used to make statistical analysis. *P<0.05.

To figure out the underlying mechanism of PrP^C^ reprogramming cholesterol metabolism, protein interactions analysis was performed between 14 cholesterol metabolism related genes (*ABCA1, SORL1, LEPR, DHCR24, ACAT2, HMGCR, IDI1, PMVK, LBR, RAN, MVK, LSS, INSIG1* and *SREBF1*) and 1086 differently expressed genes according to RNA-Sequencing results. Results showed that 6 (*APOE*, *PPARA*, *FADS2*, *FAXDC2*, *CPT1A* and *TP53*) out of 1086 genes abundantly participated in interactions with 14 cholesterol metabolism related genes, expression levels of these 6 genes were verified *in vitro* and 3 (*PPARA*, *FADS2* and *CPT1A*) of them were enriched in PPAR signaling pathway (**Figure 8A-8B, Figure S1C**), then we centered on exploring role of peroxisome proliferator-activated receptor alpha (PPARα) pathway in PrP^C^-mediated cholesterol metabolism reprogramming. Being in consistent with results of RNA-Sequencing, expression of PPARα was obviously inhibited by PrP^C^ in ESC, and down-regulating PrP^C^ had an adverse effect (**Figure 8C**).

Besides, PPARα agonist gemfibrozil encouraged cholesterol efflux related genes expression (ABCA1 and ABCG2) and decreased expression of cholesterol biosynthesis related genes, and knocking down PPARA level in ESC showed adverse effects (**Figure 8D-8F**). Furthermore, gemfibrozil promoted efflux of cholesterol and decreased cellular cholesterol concentration in ESC, and down-regulating PPARA in ESC decreased cholesterol efflux and encouraged cellular cholesterol accumulation (**Figure 8G-8H**), which provided more materials for estrogen biosynthesis, indicating that PrP^C^ reprogrammed cholesterol and estrogen biosynthesis through hindering PPARα pathway.

## Discussion

Herein, we concerned at revealing status of lipid metabolism and for the first time demonstrated a disordered cellular cholesterol metabolic state in EMs loci, which was substantially different from the condition presented in peripheral blood, and cholesterol transcriptome was indeed characterized by inhibited biosynthesis and activated efflux, transportation activities, which was unexpectedly inconsistent with our anticipations, for we found out obviously accumulated cellular cholesterol in ectopic lesions and ESC, indicating that EMs loci possessed more cholesterol supply than normal endometrium and lipid metabolic pattern dramatically altered. As to the unexpected transcriptome, the most possible reason explaining for it is that long-term continuous excess cholesterol accumulation may induce negative feedback regulation in EMs loci and encourage cholesterol lowering activities such as restricted production, accelerated efflux and enhanced application to synthesize estrogen. Additionally, our study mainly concentrated on exploring role of cellular cholesterol in encouraging estrogen production, and other biological functions of cholesterol independent of estrogen in regulating ESC behavior and EMs development was not investigated. It's reported that cholesterol could serve as a ligand of ERRα and favour cellular proliferation and facilitate metastasis behaviour by working on immune cells in breast cancer 26-27, and it hinders invasion and metastasis of hepatocellular carcinoma by means of facilitating CD44 localized in lipid raft 28, which may also matter in EMs, for cholesterol lowering drug pravastatin actually mitigated lesions in mouse models, and multiple undiscovered roles of cholesterol remain to be unearthed.

Our study confirmed activated local estrogen biosynthesis in EMs, and to exclude the possibility that this phenomenon results from distinction among tissue types, for chocolate cyst wall specimens we used are ovary derived, we analyzed transcriptome of estrogen biosynthesis related enzymes in 9 EMs related GEO datasets, in which most types of EMs are covered, and they consistently displayed the same situation in lesions, implying that milieu of EMs loci is estrogen biosynthesis encouraged, independent of lesion location, which are also proposed by some researches 29-31. As to the underlying mechanism behind this result, we verified that local cellular cholesterol accumulation made great contributions to it, denoting that excess lipid provision probably facilitates estrogen biosynthesis. Based on this theory, surroundings of EMs loci may definitely important, and we wonder whether there are adipose tissues or excess lipids deposited around the lesions, thus it is meaningful for gynecologists pay attention to the real situation during surgery, and intervention targeted on adipose and lipids may emerge as one creative approach for EMs treatment if this theory works. Except adequate material supply, other factors may also dedicate to activated local estrogen biosynthesis, like TNF-α-mediated PGC-1α accelerates local estrogen production through modulating aromatase expression and activity in ovarian EMs, reported by Izumi Suganuma 10, and Qiuming Qi puts forward that platelets favour estrogen formation of ESC through NF-κB/TGF-β1 pathway 32.

EMs loci is not only featured by estrogen biosynthesis encouraged, but also triggers estrogen-lesions-estrogen vicious circle and a local positive feedback loop operation, and we found out the critical role of PrP^C^ in this process, which is less researched and explored in gynecological disease. Our study discovered that PrP^C^ was regulated by estrogen stimulation and it promoted estrogen biosynthesis in return at the same time, implying that tight interaction exists between PrP^C^ and female reproductive system. Octapeptide repeat region is a special structure for PrP^C^ 33-34, whose importance has been verified by substantial researches, and we found out that both solely knockout this region and conventional depletion of PRNP all extensively alleviated disease severity in mouse models, hinting that this was a critical and functional region for PrP^C^ playing a role in EMs, then octapeptide repeat region of PrP^C^ may be a more ideal target for EMs intervention. Moreover, elevated PrP^C^ promoted proliferative, migratory and invasive abilities of ESC possibly via encouraging cholesterol accumulation and estrogen biosynthesis, and accumulating research has identified that estradiol enhanced invasion of ESC through regulating hsa-circ-0001649 expression 35-37 and cholesterol homeostasis influenced proliferation of bladder cancer cells 38. As to the potential mechanism of PrP^C^ reprogramming cholesterol metabolism, we mainly focused on investigating interactions among proteins, for PrP^C^ was characterized by generally taking participation in biological processes through binding to other molecules, and we found out PrP^C^-mediated PPARα pathway frequently interacted with cholesterol metabolism. Meanwhile, there is also other possibility that PrP^C^ indirectly interacts with PPARα, for Malabendu Jana reports that PPARα agonist gemfibrozil inhibits PrP^C^ expression via PI3K pathway 39. Over the last few years, PPARα pathway has been largely reported by researchers for its role in regulating lipid metabolism 40-41 and we also substantiated its role in mediating cellular cholesterol metabolism in EMs. Based on the facts that some PPARα-target chemical compounds are used as lipid lowering drugs 42-43, it is of great significance to take it into consideration that PPARα-target chemicals are applied to treat EMs patients in clinic.

Based on the previously unraveling mechanism of EMs progression verified in our study, we provided several innovate and potential approaches for EMs intervention, while there also exist many unsolved problems needed to be largely improved. For example, clinical specimens used in our study were mostly ovarian EMs, other types of specimens were rarely collected, then our results might probably only work in ovarian EMs, then more types of specimens such as adenomyosis and deep infiltrating EMs lesions need to be investigated in the future. Besides, gland made up of endometrial epithelial cells (EECs) are the main functional region of endometrium, while the primary EECs were hard to isolated and subcultured, then study on them was largely hampered, thus researchers might pay more attention to the isolation and culture of EECs, as well as endometrial organoids in the future, which could be benefit to EMs research.

**Figure 9:**
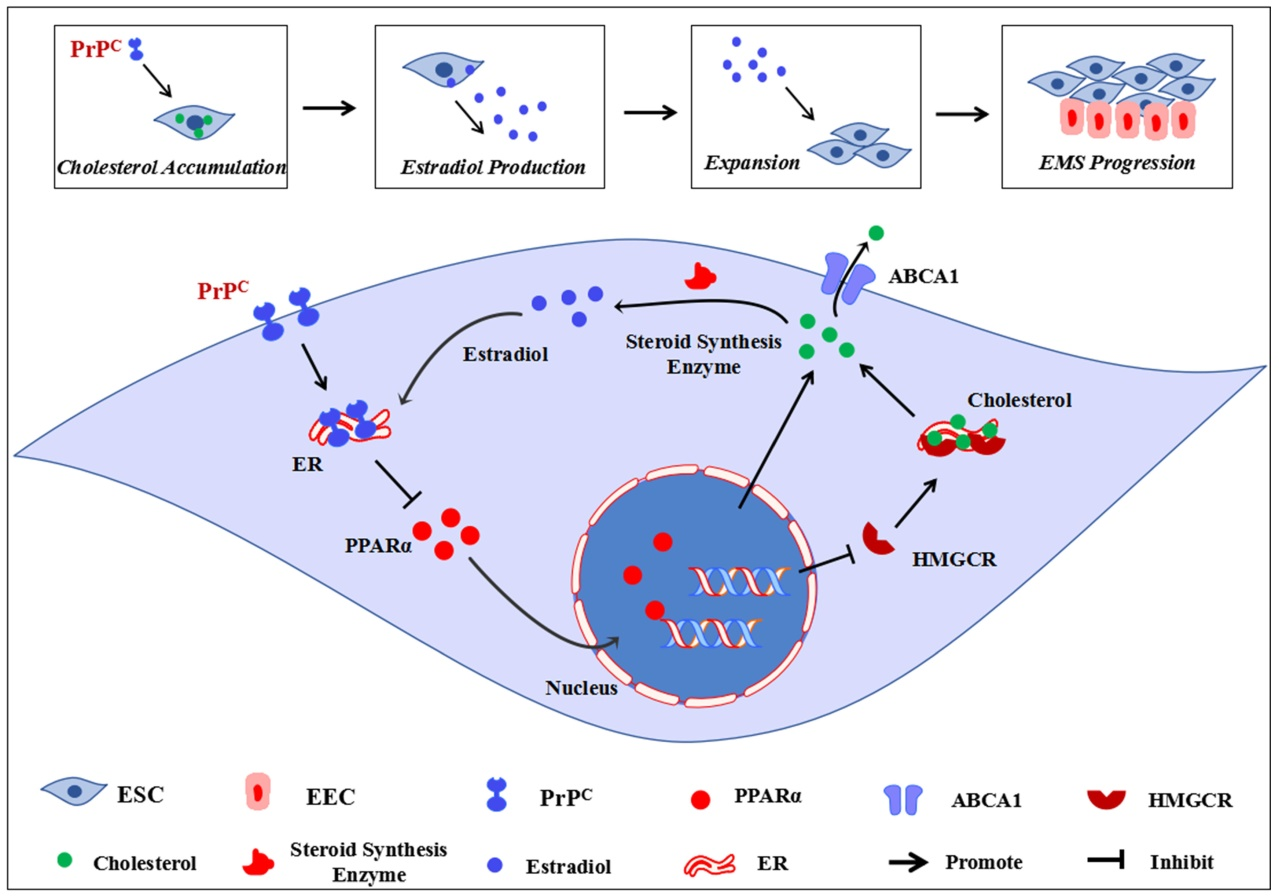
** Ideograph for demonstrating molecular mechanism of PrP^C^ promoting Ems progression.** EMs is an estrogen-dependent disease, the ectopic lesions demonstrates a state of disordered cholesterol metabolism, and the accumulated cellular cholesterol activates the steroid biosynthesis process, thus augmented local estradiol level promotes PrP^C^ expression. Up-regulating expression of PrP^C^ hinders PPARα expression, which decreases ABCA1-mediated cholesterol efflux and increases HMGCR-mediated cholesterol biosynthesis, further promotes cellular cholesterol accumulation and estradiol biosynthesis, thus promotes ESC proliferation, migration and invasion and encourages EMs progression.

Conclusively, our study primarily demonstrates that disordered cholesterol metabolism and activated estrogen biosynthesis states exist in EMs lesions, and high local estrogen level derived from ectopic ESC induces augmented expression of PrP^C^, which serves as a critical mediator in encouraging cholesterol accumulation and estrogen production through negatively regulating PPARα pathway, ultimately promoting EMs progression (**Figure 9**). Novel roles of cellular cholesterol and PrP^C^ are presented in our study, and cholesterol pharmacological inhibition strategies, PPARα targeted and genetic ablation of PrP^C^ approaches could be taken into consideration as EMs treatment methods in the future.

## Supplementary Material

Supplementary figure and tables.

## Figures and Tables

**Table 1:** Clinical characteristics of participants.

	Total samples		Samples for isolating ESC		Samples used for IHC ^a^	
**Samples**	EMs (n=29)	Control ^b^ (n=20)	eESCs (n=13)	nESCs (n=15)	Ectopic endo ^c^ (n=16)	Normal endo (n=12)
**Age (Year) ^d^**	33.52 ± 1.207	32.40 ± 1.266	35.23 ± 2.155	32.80 ± 1.418	32.13 ± 1.274	32.42 ± 1.549
**Menstrual cycle phase**						
Proliferative	24	18	10	13	14	10
Secretory	5	2	3	2	2	2
**rASRM Stage^ e^**						
I-II	7		4		3	
III-IV	22		9		13	
**Dysmenorrhea**						
No	14	16	8	11	10	11
Yes	15	4	5	4	6	1

^a^ IHC: immunohistochemistry; ^b^ Control: infertility patients were used as control patients; ^c^ endo: endometrium; ^d^ Age (Y): mean ± standard error of the mean (SEM); ^e^ rASRM: Revised classification by American Society for Reproductive Medicine.

## Abbreviations

EMs: endometriosis
*Prnp^-/-^*, KO119: PRNP knockout mouse
KO120: octapeptide repeat region of PRNP knockout mouse
Tg20: PRNP transgenic mouse
PrPC: prion, encoded by PRNP
ESCs: endometrial stromal cells
HMGCR: HMG-CoA reductase
ABCA1: ATP-binding cassette transporter type A1
ACAT1: acetyl-CoA acetyltransferase 1
PrP^SC^: misfolded isoform of prion protein
HESC: human endometrial stromal cell line
GEO: Gene Expression Omnibus database
