# Supplementary material for: PrPC Promotes Endometriosis Progression by Reprogramming Cholesterol Metabolism and Estrogen Biosynthesis of Endometrial Stromal Cells through PPARα Pathway
Source: Int J Biol Sci. 2022 Feb 7;18(4):1755–72. doi: 10.7150/ijbs.68015 (PMC8898366; doi:10.7150/ijbs.68015)
Supplement: Supplementary file 1 — Supplementary figure and tables. [file ijbsv18p1755s1.pdf]

# Supplementary information

# PrP$^{C}$ Promotes Endometriosis Progression By Reprogramming Cholesterol Metabolism And Estrogen Biosynthesis Of Endometrial Stromal Cells Through PPARα Pathway

Hai-Yan Peng, Sha-Ting Lei, Shu-Hui Hou, Li-Chun Weng, Qing Yuan, Ming-Qing Li, Dong Zhao

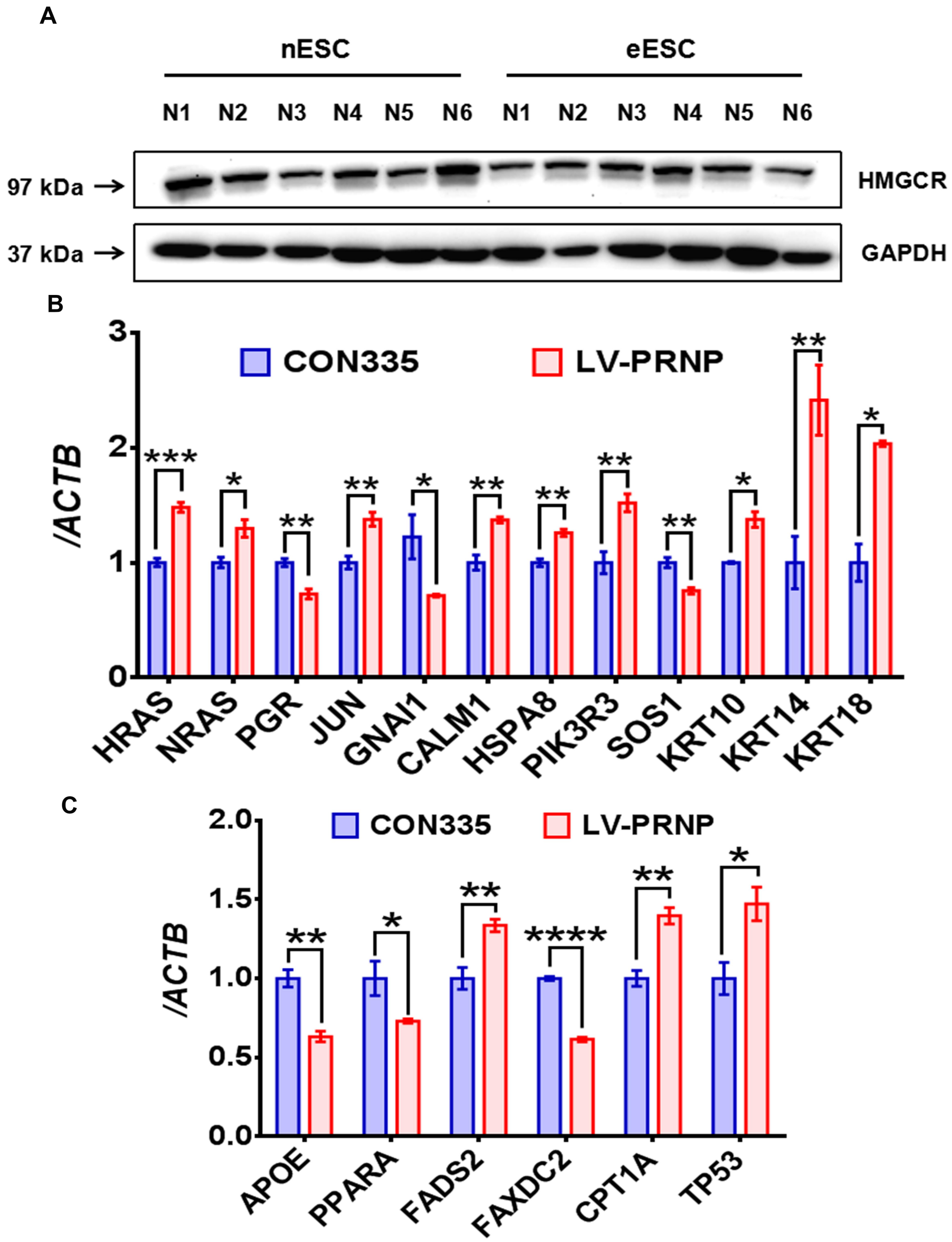


**Figure S1: Verification of the next RNA-Sequencing results.** (A) Protein level of HMGCR decreased in eESC, when compared to nESC. (B) Expression levels of estrogen signaling pathway related genes were verified by qRT-PCR assay. (C) Expression of *APOE*, *PPARA*, *FADS2*, *FAXDC2*, *CPT1A* and *TP53* were verified. Data was showed as mean ± SEM, Student's t-test, one-way analysis of variance (ANOVA) and Mann-Whitney test were used to make statistical analysis. *P<0.05, **P<0.01, ***P<0.001, ****P<0.0001.

**Table S1: The clinical information of patients in the study.**

| No. | Diagnosis | Specimens | Age | Cycle Phase | Dysmenorrhea | rASRM Stage | Assays used |
|---|---|---|---|---|---|---|---|
| 1 | Ovarian EMs | EEM, EESC | 31 | Proliferative | Yes | III | qRT-PCR, ELISA |
| 2 | Ovarian EMs | EEM, EESC | 25 | Secretory | no | IV | qRT-PCR, ELISA |
| 3 | Ovarian EMs | EEM, EESC | 37 | Proliferative | no | II | qRT-PCR, ELISA |
| 4 | Ovarian EMs | EEM, EESC | 40 | Proliferative | no | II | qRT-PCR, ELISA |
| 5 | Ovarian EMs | EEM, EESC | 46 | Secretory | no | III | qRT-PCR, ELISA |
| 6 | Ovarian EMs | EEM, EESC | 43 | Proliferative | no | III | qRT-PCR, ELISA |
| 7 | Ovarian EMs | EEM, EESC | 26 | Proliferative | Yes | II | qRT-PCR, ELISA |
| 8 | Ovarian EMs | EEM, EESC | 39 | Secretory | no | II | qRT-PCR, ELISA |
| 9 | Ovarian EMs | EEM, EESC | 24 | Proliferative | Yes | III | qRT-PCR, ELISA |
| 10 | Ovarian EMs | EEM, EESC | 33 | Proliferative | Yes | III | qRT-PCR, WB, ELISA |
| 11 | Ovarian EMs | EEM, EESC | 39 | Proliferative | no | III | qRT-PCR, WB, ELISA |
| 12 | Ovarian EMs | EEM, EESC | 46 | Proliferative | no | III | qRT-PCR, WB, ELISA |
| 13 | Ovarian EMs | EEM, EESC | 29 | Proliferative | Yes | III | qRT-PCR, WB, ELISA |
| 14 | Ovarian EMs | EEM | 40 | Proliferative | Yes | III | WB, IHC |
| 15 | Ovarian EMs | EEM | 31 | Proliferative | Yes | IV | WB, IHC |
| 16 | Ovarian EMs | EEM | 37 | Proliferative | no | III | WB, IHC |
| 17 | Ovarian EMs | EEM | 39 | Proliferative | no | III | WB, IHC |
| 18 | Ovarian EMs | EEM | 32 | Proliferative | no | III | WB, IHC |
| 19 | Ovarian EMs | EEM | 27 | Proliferative | no | III | WB, IHC |
| 20 | Ovarian EMs | EEM | 29 | Secretory | no | III | WB, IHC |
| 21 | Ovarian EMs | EEM | 43 | Proliferative | Yes | IV | WB, IHC |
| 22 | Ovarian EMs | EEM | 31 | Proliferative | Yes | III | WB, IHC |
| 23 | Ovarian EMs | EEM | 25 | Proliferative | Yes | II | WB, IHC |
| 24 | Ovarian EMs | EEM | 30 | Proliferative | no | III | WB, IHC |
| 25 | Ovarian EMs | EEM | 29 | Proliferative | Yes | III | WB, IHC |
| 26 | Ovarian EMs | EEM | 34 | Proliferative | Yes | II | WB, IHC |

| | | | | | | | |
|---|---|---|---|---|---|---|---|
| 27 | Ovarian EMs | EEM | 30 | Proliferative | Yes | II | WB, IHC |
| 28 | Ovarian EMs | EEM | 29 | Secretory | Yes | III | WB, IHC |
| 29 | Ovarian EMs | EEM | 28 | Proliferative | Yes | III | WB, IHC |
| 30 | Tubal Infertility | NEM, NESC | 31 | Proliferative | no | - | qRT-PCR, ELISA, IHC |
| 31 | Tubal Infertility | NEM, NESC | 26 | Proliferative | no | - | qRT-PCR, ELISA, IHC |
| 32 | Tubal Infertility | NEM, NESC | 38 | Proliferative | no | - | qRT-PCR, ELISA, IHC |
| 33 | Tubal Infertility | NEM, NESC | 36 | Secretory | no | - | qRT-PCR, ELISA, IHC |
| 34 | Tubal Infertility | NEM, NESC | 35 | Proliferative | no | - | qRT-PCR, WB, ELISA |
| 35 | Tubal Infertility | NEM, NESC | 30 | Proliferative | Yes | - | qRT-PCR, WB, ELISA |
| 36 | Tubal Infertility | NEM, NESC | 29 | Proliferative | no | - | qRT-PCR, WB, ELISA |
| 37 | Tubal Infertility | NEM, NESC | 30 | Proliferative | Yes | - | qRT-PCR, WB, ELISA |
| 38 | Tubal Infertility | NEM, NESC | 40 | Proliferative | no | - | qRT-PCR, WB, ELISA |
| 39 | Tubal Infertility | NEM, NESC | 33 | Proliferative | no | - | qRT-PCR, WB, ELISA |
| 40 | Tubal Infertility | NEM, NESC | 41 | Proliferative | no | - | qRT-PCR, WB, ELISA |
| 41 | Tubal Infertility | NEM, NESC | 21 | Proliferative | Yes | - | qRT-PCR, WB, ELISA |
| 42 | Tubal Infertility | NEM, NESC | 37 | Secretory | no | - | WB, ELISA, IHC |
| 43 | Tubal Infertility | NEM, NESC | 29 | Proliferative | Yes | - | WB, ELISA, IHC |
| 44 | Tubal Infertility | NEM, NESC | 36 | Proliferative | no | - | WB, ELISA, IHC |
| 45 | Tubal Infertility | NEM | 41 | Proliferative | no | - | qRT-PCR, WB, IHC |
| 46 | Tubal Infertility | NEM | 25 | Proliferative | no | - | qRT-PCR, WB, IHC |
| 47 | Tubal Infertility | NEM | 28 | Proliferative | no | - | qRT-PCR, WB, IHC |
| 48 | Tubal Infertility | NEM | 27 | Proliferative | no | - | qRT-PCR, WB, IHC |
| 49 | Tubal Infertility | NEM | 35 | Proliferative | no | - | WB, IHC |

**Note:**

No.: Number; EEM: ectopic endometrium; NEM: normal endometrium; EESC: ectopic endometrial stromal cells; NESC: normal endometrial stromal cells; qRT-PCR: quantitative real-time PCR assay; WB: western blotting assay; IHC: immunohistochemistry; ELISA: enzyme-linked immunosorbent assay.

**Table S2: Primers for qRT-PCR assay.**

| Gene Name | Forward or Reverse | Sequence |
|---|---|---|
| Human stAR | Forward | GGCATCCTTAGCAACCAAGA |
| Human stAR | Reverse | TCTCCTTGACATTGGGGTTC |
| Human CYP17A1 | Forward | TCTCTGGGCGGCCTCAA |
| Human CYP17A1 | Reverse | AGGCGATACCCTTACGGTTGT |
| Human CYP19A1 | Forward | GACTTTGCCACTGAGTTGATTT |
| Human CYP19A1 | Reverse | CGATCAGCATTTCCAATATGCA |
| Human CYP11A1 | Forward | GAGATGGCACGCAACCTGAAG |
| Human CYP11A1 | Reverse | CTTAGTGTCTCCTTGATGCTGGC |
| Human HSD3B2 | Forward | CAGGCTCTTTTCAGGAATGG |
| Human HSD3B2 | Reverse | CTTGGACAAGGCCTTCAGAC |
| Human PGR | Forward | CCCAGCATGTCGCCTTAGAAA |
| Human PGR | Reverse | AGTGCTCTCACAACTCTGACTT |
| Human B-Actin | Forward | TGGCACCCAGCACAATGAA |
| Human B-Actin | Reverse | CTAAGTCATAGTCCGCCTAGAAGCA |
| Human GAPDH | Forward | GCCTCAAGATCATCAGCAATGCCT |
| Human GAPDH | Reverse | GTGGTCATGAGTCCTTCCACGAT |
| Human HSD17B7 | Forward | AAGGCAAGGAACCCTACAGCTCTT |
| Human HSD17B7 | Reverse | AATATTGCCGGCATCAACAGCGTC |
| human PRNP | Forward | AATCAAGCAGCACACGGTCA |
| human PRNP | Reverse | TCGGTGAAGTTCTCCCCCTT |
| Human ACAT2 | Forward | GAGACTTACCCTAGGACGCCCT |
| Human ACAT2 | Reverse | AGTTCTTGGCCACATAATTCCAC |
| Human HMGCS1 | Forward | AAAGTACCAAGACTCCCTGCCACA |
| Human HMGCS1 | Reverse | ATCCCATTCCTCCAACTGTTCCCA |
| Human HMGCR | Forward | GCCTGGCTCGAAACATCTGAA |
| Human HMGCR | Reverse | CTGACCTGGACTGGAAACGGATA |
| Human SQLE | Forward | CTATGGCAGAGCCCAATGCAAAGT |

| | | |
|---|---|---|
| Human SQLE | Reverse | ACAACAGTCAGTGGAGCATGGAGT |
| Human DHCR24 | Forward | GTGAAACACTTTGAAGCCAGG |
| Human DHCR24 | Reverse | AGCCATCAAACATCTCCCAG |
| Human IDI1 | Forward | GCATCGAGCTTTTAGTGTCTTCT |
| Human IDI1 | Reverse | GGCTGGATTGCTTAATGGATGA |
| Human DHCR7 | Forward | CAGCGCCAGAGACTGCAAA |
| Human DHCR7 | Reverse | TGAAGAACAGCTTGAAGTCAAACC |
| Human LSS | Forward | CATCAACATGCTTGTGCGCTGGTA |
| Human LSS | Reverse | ATTTTCATGCCGTCAAGGCCCATC |
| Human SREBP-1 | Forward | CCCATGGGAGAGCCTGTACA |
| Human SREBP-1 | Reverse | CTAAGAGATGTTCCCGGAATAGCT |
| Human SREBP-2 | Forward | ACAACCCATAATATCATTGAGAAACG |
| Human SREBP-2 | Reverse | TTGTGCATCTTGGCGTCTGT |
| Human CYP51 | Forward | GAGTGATGCTGCTGCACTGCTTTT |
| Human CYP51 | Reverse | ACATCGTATGCAACTCCCTTCCCA |
| Human ACAT1 | Forward | GCAGCGAAGAGGCTCAATG |
| Human ACAT1 | Reverse | GCAGCATATACAGGAGCAATTGG |
| Human ABCG2 | Forward | TTTCCAAGCGTTCATTCAAAAA |
| Human ABCG2 | Reverse | TACGACTGTGACAATGATCTGAGC |
| Human ABCA1 | Forward | ATGTCCAGTCCAGTAATGGTTCTGT |
| Human ABCA1 | Reverse | CGAGATATGGTCCGGATTGC |
| Human APOE | Forward | TGCCCGGCCTCCTAGCTCCTTC |
| Human APOE | Reverse | GAGGTGAAGGAGCAGGTGGCGGAG |
| Human PPARA | Forward | GGCGAGGATAGTTCTGGAAGC |
| Human PPARA | Reverse | CACAGGATAAGTCACCGAGGAG |
| Human FADS2 | Forward | TCATGACCATGATCGTCCATAAGAA |
| Human FADS2 | Reverse | GCTCCCAGGATGCCGTAGAA |
| Human FAXDC2 | Forward | GACATGGTGGATCCTGTGAAACT |
| Human FAXDC2 | Reverse | GAAAGATATCATGCACTGGTTGAAA |

| | | |
|---|---|---|
| Human CPT1A | Forward | ATCAATCGGACTCTGGAAACGG |
| Human CPT1A | Reverse | TCAGGGAGTAGCGCATGGT |
| Human TP53 | Forward | CCTGGATTGGCCAGACTGC |
| Human TP53 | Reverse | TTTTCAGGAAGTAGTTTCCATAGGT |
| Human HRAS | Forward | AGAGGATTCCTACCGGAAGCAGGT |
| Human HRAS | Reverse | TGCTCCCTGTACTGGTGGATGT |
| Human NRAS | Forward | CCTCTACAGGGAGCAGATTAAGCGA |
| Human NRAS | Reverse | CCCTGTCTGGTCTTGGCTGAGGT |
| Human JUN | Forward | AGGAAGCTGGAGAGAATCGC |
| Human JUN | Reverse | GTTAGCATGAGTTGGCACCC |
| Human GNAI1 | Forward | ACGGACACATCCATCATCCT |
| Human GNAI1 | Reverse | TCCGTTACAGCATCGAACAC |
| Human CALM1 | Forward | ACAAGGATGGGAATGGTTACAT |
| Human CALM1 | Reverse | TGCAGTCATCATCTGTACGAAT |
| Human HSPA8 | Forward | CACCAACACAGTTTTTGATGCCA |
| Human HSPA8 | Reverse | GATGAGCACGTTTCTTTCTGCTC |
| Human PIK3R3 | Forward | TACAATACGGTGTGGAGTATGGA |
| Human PIK3R3 | Reverse | TCATTGGCTTAGGTGGCTTTG |
| Human SOS1 | Forward | GGAGATCAACCCTTGAGTGCAG |
| Human SOS1 | Reverse | TGCTCTACCCAGTGCCGACATA |
| Human KRT10 | Forward | CTTGGCAGAAACAGAAGGTCGCT |
| Human KRT10 | Reverse | GCAGGCTGCGGTAGGTTTGAA |
| Human KRT14 | Forward | GGCCTGCTGAGATCAAAGACTAC |
| Human KRT14 | Reverse | CACTGTGGCTGTGAGAATCTTGTT |
| Human KRT18 | Forward | GCTGACCGACGAGATCAACT |
| Human KRT18 | Reverse | CATGGACAGCACCACAGATG |

**Table S3: Reagents used in this study.**

| Reagent | Application | Manufacturer | Catalogue |
|---|---|---|---|
| Anti-prion | WB/IHC | Abcam | ab52604 |
| Anti-HMGCR | WB | abcam | Ab174830 |
| Anti-ABCA1 | WB | abcam | Ab18180 |
| Anti-CYP19A1 (Aromatase) | WB | CST | 14528 |
| Anti-CYP17A1 | WB | abcam | Ab125022 |
| Anti-CYP11A1 | WB | abcam | Ab175408 |
| Anti-PCNA | WB | CST | 13110 |
| Anti-PPARα | WB | abcam | Ab215270 |
| Anti-GAPDH | WB | CST | 5174S |
| Anti-Actin | WB | CST | 4970 |
| PE anti-human CD230 (Prion) | FCM | Biolegend | 800318 |
| DAB HRP Color Development Kit | IHC | Biotech Well | WB0167 |
| RIPA | WB | Biotech Well | WB0101 |
| 4% Paraformaldehyde | Reagent | Biotech Well | WH1011 |
| PVDF membrane | WB | Millipore | IPVH00010 |
| CELLSAVING | Cell Culture | NCM | C40050 |
| Pravastatin | Reagent | MedChemExpress | HY-B0165A |
| Gemfibrozil | Reagent | MedChemExpress | HY-B0258 |
| 17-β estradiol | Reagent | Sigma | E2758 |
| PrimeScript RT Master Mix | qRT-PCR | Takara | RR036A |
| TB Green Premix Ex TaqTMII | qRT-PCR | Takara | RR820A |
| RNAiso Plus | qRT-PCR | Takara | 9109 |
| 17-β estradiol ELISA Kit | ELISA | IBL | RE52041 |
| Amplex Red Cholesterol Assay Kit | Reagent | Invitrogen | A12216 |
| Cholesterol Assay Kit (Cell-Based) | Reagent | abcam | Ab133116 |
| U18666A | Reagent | abcam | Ab133116 |
| Immobilon Western Chemilum HRP | WB | Millipore | WBKLS0100 |

| | | | |
|---|---|---|---|
| Cell Counting Kit-8 | Reagent | DOJINDO | CK04 |
| Matrigel | Reagent | Corning | 356234 |
| DMSO | Reagent | Sigma | D8418 |
